# Effect of Protective Measures Adopted in the COVID-19 Pandemic on Hemodialysis Patients

**DOI:** 10.7759/cureus.35552

**Published:** 2023-02-27

**Authors:** Duarte A Ferreira, Carlota Vida, Érica Mendonça, Pedro Vieira, Sónia Freitas, Gil Silva

**Affiliations:** 1 Internal Medicine, Hospital Central do Funchal, Funchal, PRT; 2 Nephrology, Hospital Central do Funchal, Funchal, PRT; 3 Family Medicine, Hospital Central do Funchal, Funchal, PRT; 4 Research, Centro de Investigação Dra. Maria Isabel Mendonça, Hospital Central do Funchal, Funchal, PRT

**Keywords:** covid-19, sars-cov2, lower respiratory infections, hemodialysis, airway protective measures

## Abstract

Introduction

The use of masks and other preventive measures is nowadays an essential measure to prevent COVID-19 infections, particularly in hemodialysis patients. The aim of this study was to understand whether these protective measures adopted during the COVID-19 pandemic reduced or somehow contained the number of respiratory infections in a population of hemodialysis patients.

Methods

This was a longitudinal retrospective single-center study of hemodialysis patients with at least six months of follow-up in a central hospital. A total of 103 patients were evaluated for the study. Two groups were defined: a control group that was followed in the year before the beginning of the pandemic and a group that followed in the year after its beginning.

Results

Patients in the pandemic group had a higher prevalence of previous major cardiovascular events (48.9% vs 8.6%) and heart failure (31.3% vs 12.1%) than those in the control group. Vaccination rates for influenza and pneumococcus as well as the monthly analytical results were similar in both groups. There were no significant differences in lower respiratory infections, hospitalizations caused by lower respiratory infections, and mortality between both groups. However, not accounting for aspiration pneumonias, the pandemic group had half the mortality due to respiratory infections (2.2% vs 5.2%).

Conclusion

Despite patients in the pandemic group having a similar prevalence of respiratory infections and hospitalizations motivated by lower respiratory infections, they presented about half the mortality of the control group. This suggests that although there was no decrease in the number of infections, protective measures may have contributed to a decreased mortality.

## Introduction

The utilization of preventive measures, such as masks, by staff and patients, greater distance between each patient during hemodialysis, and the use of individual protection equipment by nurses was not a common practice in hemodialysis units. However, there is now evidence that these preventive measures could decrease the incidence of other respiratory infections beyond COVID-19 [1].

With the beginning of the COVID-19 pandemic, several measures were adopted. Madeira Island, due to its geographical characteristics and particularities, such as having its airport closed at the beginning of the pandemic, represented an excellent place to reach conclusions without external constraints. Along with the lesser circulation of airborne COVID-19 in the local population, these measures could also represent a protective factor against other respiratory infections besides COVID-19.

Furthermore, during hemodialysis sessions, the patients started to be placed at least two meters away from each other, wearing a surgical mask became mandatory, frequent hand hygiene was promoted, breaks for food were suspended during sessions, patients were contacted before each session in order to track any symptoms or high-risk contacts, and transportation to the hospital was guaranteed individually for each patient. Isolation measures were established for patients confirmed to be infected or for those signaled as high-risk contacts. Besides, despite being asymptomatic, patients and all the staff working in the hospital unit were submitted to a PCR SARS-CoV2 screening every 15 days.

The main goal of this study was to understand whether the protective measures adopted with COVID-19 could bring any benefit in terms of preventing other respiratory infections in hemodialysis patients. Thus, the objective was to comprehend whether, even after the end of the pandemic, these measures should be maintained in order to improve the outcomes of these patients.

## Materials and methods

This is a longitudinal retrospective, single-center study of hemodialysis patients followed in a central hospital.

Two groups were defined, a control group with hemodialysis patients followed in a hospital environment in the year before the beginning of the pandemic (April 2019 to March 2020) and a group with hemodialysis patients followed in the year after the beginning of the pandemic (April 2020 to March 2021). These time limits were established since COVID-19 cases only started to be reported by the end of March 2020, given the geographical specificity of Madeira Island and the public health measures assumed.

Only patients with a hospital follow-up of more than six months were considered. All those who did not meet this requirement were excluded.

We used demographic variables including sex and age, and evaluated comorbidities such as hypertension, diabetes, and dyslipidemia; major cardiovascular events history (CVH) accounting for nonfatal stroke and nonfatal myocardial infarction; heart failure; chronic liver disease; malignancy diseases; human immunodeficiency virus (HIV); or concomitant hepatotropic virus infection (hepatitis B virus [HBV] and hepatitis C virus [HCV]). Previous vaccination for influenza, pneumococcus,* *or COVID-19 (the latter in the post-pandemic group) was documented. Monthly analytical data from these patients were also collected, with particular emphasis on hemoglobin, calcium, phosphorus, parathyroid hormone (PTH), albumin, and ferritin as part of the routine assessment of hemodialysis patients.

Finally, the existence of lower respiratory infections, those leading to hospital admissions, morbidity, and mortality due to respiratory infections, was documented. Data were analyzed using Statistical Package for Social Sciences (SPSS), version 27.0 (IBM Corp., Armonk, NY).

## Results

A total of 103 patients were evaluated for the study, with 58 in the control group and 45 in the post-pandemic group. There were no significant differences in gender distribution (p-value = 0.611) or age (p-value = 0.624). Regarding comorbidities (Table 1), patients in the pandemic group had an almost six-fold higher prevalence of CVH than those in the control group (48.9% vs 8.6%; p-value < 0.0001) and had an almost three-fold higher heart failure (31.3% vs 12.1%; p-value = 0.017). Malignancy was also higher in the pandemic group (22.2% vs 13.8%), but the difference was not statistically significant. As for the remaining comorbidities, such as diabetes and arterial hypertension, they were similar in both groups.

**Table 1:** Patient's characteristics, analytical study, hospital admissions, and deaths ANCA: Antineutrophilic cytoplasmic antibody; AV: Arteriovenous; CVH: Cardiovascular events history; HBV: Hepatitis B virus; HCV: Hepatitis C virus.

	Control group	Post-pandemic group	P-value	
Number of patients	58	45		
Age (years)	61.1 (± 17.9)	62.8 (± 15.1)	0.624	
Sex				
Male	30 (51.7%)	21 (46.7%)	0.611	
Female	28 (48.3%)	24 (53.3%)		
Chronic kidney disease etiology				
Diabetes	27 (46.6%)	20 (44.4%)		
Arterial hypertension	6 (10.3%)	2 (4.4%)		
Obstructive nephropathy	6 (10.3%)	5 (11.1%)		
Reflux nephropathy	2 (3.4%)	0 (0.0%)		
Chronic pyelonephritis	2 (3.4%)	2 (4.4%)		
Cardio-renal syndrome	1 (1.7%)	1 (2.2%)		
Autosomal dominant polycystic kidney disease	2 (3.4%)	1 (2.2%)		
IgA nephropathy	2 (3.4%)	2 (4.4%)		
Membranous glomerulonephritis	1 (1.7%)	1 (2.2%)		
Focal segmental glomerulosclerosis	0 (0.0%)	1 (2.2%)		
ANCA vasculitis	0 (0.0%)	1 (2.2%)		
Thrombotic microangiopathy	1 (1.7%)	0 (0.0%)		
Lupus nephritis	1 (1.7%)	1 (2.2%)		
Cast nephropathy	1 (1.7%)	1 (2.2%)		
Non-steroidal anti-inflammatory drugs nephropathy	0 (0.0%)	1 (2.2%)		
Unknown	6 (10.3%)	6 (13.3%)		
Vascular access (%)				
AV fistula	21 (36.2%)	11 (24.4%)		
AV graft	2 (3.4%)	2 (4.4%)		
Catheter	35 (60.4%)	32 (71.1%)		
Comorbid conditions				
Diabetes	30 (51.7%)	26 (57.8%)	0.541	
Hypertension	32 (55.2%)	28 (62.2%)	0.472	
Dyslipidemia	6 (10.3%)	2 (4.4%)	0.461	
CVH	5 (8.6%)	22 (48.9%)	<0.0001	
Heart failure	7 (12.1%)	14 (31.1%)	0.017	
Chronic liver disease	0	2 (4.4%)	0.188	
Neoplastic diseases	8 (13.8%)	10 (22.2%)	0.264	
HBV	1 (1.7%)	0 (0.0%)	-	
HCV	3 (5.2%)	2 (4.4%)		
HIV	1 (1.7%)	1 (2.2%)		
HIV + HBV	0 (0.0%)	0 (0.0%)		
HIV + HCV	1 (1.7%)	1 (2.2%)		
Vaccination				
Influenza	25 (43.1%)	23 (51.1%)	0.419	
Pneumococcus	4 (6.9%)	4 (8.9%)	0.727	
COVID-19	-	35 (77.8%)	-	
Analytical data				Reference values
Hemoglobin (g/dL)	11.1 (± 1.0)	10.8 (± 1.3)	0.282	13.7-17.3
Calcium (mg/dL)	8.8 (± 0.7)	8.8 (± 0.8)	0.984	8.9-10.3
Phosphor (mg/dL)	4.3 (± 1.5)	4.6 (± 1.5)	0.328	2.4-4.7
Parathyroid hormone (pg/mL)	528.6 (± 824.7)	379.6 (± 711.6)	0.482	130-550
Albumin (g/L)	35.7 (± 4.7)	35.7 (± 4.5)	0.709	35-48
Ferritin (ng/mL)	583.9 (± 1383)	558.1 (± 477.7)	0.935	30-400
Lower respiratory infections	6 (10.3%)	8 (17.8%)	0.386	
Lower respiratory infections leading to hospital admissions	5 (8.6%)	8 (17.8%)	0.233	
Deaths	11 (19%)	8 (17.8%)	0.877	
Deaths due to respiratory infections	3 (5.2%)	3 (6.7%)	1.000	

Vaccination rates (Table 1) for influenza and pneumococcus were also identical, and it should be noted that by the end of the time period studied, 77.8% of patients in the pandemic group had already been vaccinated for COVID-19.

In terms of analytical results, the values were similar between both groups regarding hemoglobin, ferritin, albumin, calcium, phosphorus, and PTH concentration (Table 1). None of the above variables remained in the logistic regression equation, so none of them independently influenced mortality.

There were no significant differences in lower respiratory infections between the groups (17.8% vs 10.3%, p-value = 0.386) and in hospitalizations motivated by lower respiratory infections (17.8 vs 8.6%; p-value = 0.233). None of these respiratory infections was due to COVID-19.

Mortality was also similar between both groups (17.8% vs 19%, p-value = 0.877), with 6.7% of deaths caused by respiratory infections in the pandemic group and 5.2% in the control group (p-value = 1). Nevertheless, it should be noted that in the control group, two of the deaths by respiratory infections were due to aspiration pneumonia. In this sense, if these deaths are not accounted for (since they effectively do not reflect an airborne infection and therefore are not preventable with airway protection measures), the mortality in the pandemic group was about half the mortality of the control group (2.2% vs 5.2%).

## Discussion

Patients on hemodialysis have an increased risk of infections. This can be explained by the higher burden of comorbidities, the intrinsic frailty presented, the existence of vascular accesses, and the frequent exposure to hospital settings and other infected patients [2].

For all these reasons, hemodialysis is an important risk factor for mortality from COVID-19 [2,3]. Most patients receive in-center hemodialysis, which ensures optimal physical isolation and infection control. With the beginning of the COVID-19 pandemic, measures such as social distancing, mask-wearing, hand washing, triage, and isolation of suspected/confirmed cases had to be implemented in dialysis care delivery in order to prevent the spread of the infection.

Madeira Island has certain characteristics that make it unique for this study, not only for its geography but also for the magnitude of the protective measures taken. Besides mask-wearing, the lockdown was imposed, and the airport closed right after the first cases were detected in Portugal (in March 2020). After the airport reopened (in July 2020), all travelers had to undergo PCR tests (before boarding or on arrival), and they also had to stay in prophylactic isolation for two weeks after arrival in the initial months of the pandemic. As intended, these measures culminated in a low incidence of new COVID-19 cases, and the incidence of the disease never exceeded 50 cases per million inhabitants per day until mid-October 2020.

All of this context reinforces the comparative power of this study. Since there was no significant COVID-19 circulation (and a complete absence of cases in hemodialysis patients), it is possible to analyze the protective effects of these measures on respiratory infections other than COVID-19. These exceptional measures will hardly be imposed in the same magnitude in the near future, rendering this a unique opportunity to reflect on these issues.

However, contrary to what was expected, there were no significant differences between the pandemic and control groups regarding the prevalence of respiratory infections and hospitalizations motivated by lower respiratory infections. This is the opposite of what has been documented in the literature, with reports of lower incidences of influenza and pneumococcus infections [1,4-8].

This could be explained by the greater surveillance for respiratory symptoms in a period with still reduced cases of COVID-19, which may have contributed to the maximization of the identification of respiratory infections, a more aggressive therapeutic approach, and eventually more hospitalizations.

Another important factor is the higher prevalence of important comorbid conditions in the pandemic group, such as CVH and heart failure. This could be explained by the fact that some patients with major comorbidities were transferred from other dialysis clinics to the hospital center for stricter surveillance and more rigorous protective measures.

There are several risk factors for lower respiratory infections in adults, such as older age, chronic or structural pulmonary disease, previous lower respiratory infections, poor nutritional status, or immunosuppression [9]. On the other hand, there seems to be no influence of other factors, such as being overweight, passive smoking, and chronic renal disease [9]. The latter may support the extrapolation of these results to the remaining population, not limiting them to this specific population.

Moreover, some comorbidities are associated with worse outcomes, despite not being related to an increased incidence of pneumonia. These comorbidities have been exhaustively described recently in the COVID-19 pandemic. A relevant set of comorbidities reported are cardiovascular diseases, which are associated with worse outcomes and increased risk of death [10,11]. Therefore, previous myocardial infarction, hypertension, diabetes, renal disease, and smoking are associated with an increased likelihood of severe COVID-19 and worse outcomes [11-14]. It is also important to point out the particular case of cerebrovascular disease, which is also associated with worse outcomes in COVID-19. However, in the literature, it is generally unclear whether the stroke occurred before or after the respiratory infection [14]. Liver disease and obesity were also associated with higher mortality in COVID-19 [14].

This higher prevalence of patients with greater comorbidities in the post-pandemic group might be due to the transfer of many of the other patients with fewer comorbidities to outpatient hemodialysis units so that cohorts for COVID-19 patients could be opened in the hospital unit, and greater safety and effectiveness of protective measures for the remaining patients could be guaranteed. This selection of patients with higher comorbidities and consequent lower physiological reserve may have been a strong reason for the absence of a clear and massive difference in respiratory infections and hospitalizations motivated by lower respiratory infections. Nevertheless, neither CVH nor heart failure independently influenced mortality.

The mortality in the pandemic group was about half the mortality of the control group (if deaths from aspiration pneumonias are not accounted for). This could suggest that, although there was no decrease in the number of infections, protective measures may have contributed to a decrease in mortality. However, the low numbers and relatively low mortality in this sample do not allow us to draw definitive conclusions.

Considering that we have now more information about the virus behavior and vaccination is part of our daily practice, more studies are needed to understand the real impact of these measures and their effectiveness in this scenario. It is also worth mentioning that it is still possible to optimize vaccination against influenza and pneumococcus.

Finally, it is pertinent to highlight some of the limitations of the study, such as the limited sample size for statistical measurements, as mentioned above, and the retrospective nature of the study.

## Conclusions

Madeira Island has some characteristics that make it unique for this study. All of the exceptional measures that were adopted will hardly be imposed in the same magnitude in the near future, allowing a unique analysis of this subject. Despite patients in the pandemic group having a similar prevalence of respiratory infections and hospitalizations motivated by lower respiratory infections, they presented about half the mortality of the control group. This suggests that although there was no decrease in the number of infections, protective measures may have contributed to a decreased mortality.
